# Failure mode and effects analysis-guided digital redesign of the intraoperative frozen-section specimen pathway: a before-and-after service improvement study

**DOI:** 10.3389/fmed.2026.1955216

**Published:** 2026-10-28

**Authors:** Juan Liu, Xu Li, Huating Zhang, Shuwei Wang, Xiaoyang Zhou

**Affiliations:** Department of Anesthesiology and Perioperative Medicine, Second Division, Central Campus, Shandong First Medical University Affiliated Provincial Hospital, Jinan, China

**Keywords:** failure mode and effects analysis (FMEA), digital transformation, intraoperative frozen section, pathology workflow, quality improvement, patient safety

## Abstract

**Background:**

Intraoperative frozen-section specimen pathways cross professional and departmental boundaries and are vulnerable to identification, handover, and transport failures. A failure mode and effects analysis (FMEA)-guided closed-loop workflow redesign may provide a structured approach to improving specimen traceability and reducing preventable process failures.

**Aim:**

To evaluate an FMEA-guided digital redesign program incorporating a dedicated frozen-section pathway, standardized verification procedures, barcode-based handover, repeat-submission registration, targeted training, and real-time workflow monitoring.

**Methods:**

This single-center, non-randomized before-and-after study was conducted at a tertiary teaching hospital in eastern China. All registered frozen-section specimens from October–December 2025 (pre-implementation; *n* = 238) and January–March 2026 (post-implementation; *n* = 247) were included. The intervention included a dedicated frozen-section pathway, standardized verification procedures, a single-record register for repeat submissions, barcode-based handover, role-specific training, and real-time monitoring of workflow deviations.

**Results:**

Transport timeliness improved from 80.7% (192/238) before implementation to 98.4% (243/247) after implementation, representing an absolute increase of 17.7 percentage points (95% CI 12.5–23.0; risk ratio 1.22, 95% CI 1.14–1.30; *p* < 0.001). Handover-record accuracy improved from 75.2% (179/238) to 99.2% (245/247), representing an absolute increase of 24.0 percentage points (95% CI 18.4–29.6; risk ratio 1.32, 95% CI 1.23–1.42; *p* < 0.001). Staff-reported satisfaction increased descriptively from 71.0% (44/62) to 95.2% (59/62).

## Highlights

- Intraoperative frozen-section specimen management requires coordinated control across the operating theater, transport service, and pathology department.
- Failure mode and effects analysis identified four priority risks involving specimen information, labeling, handover documentation, and transport timeliness.
- A dedicated digital pathway combined barcode-based handover, repeat-submission registration, role-specific training, and real-time monitoring.
- The program was associated with substantial improvements in transport timeliness and handover-record accuracy.
- The program illustrates the coordinating role that perioperative nurses can play in end-to-end specimen traceability and multidisciplinary service improvement.

## Introduction

Intraoperative rapid pathology, commonly known as frozen-section examination, is an essential diagnostic procedure during surgery, providing surgeons with timely information regarding lesion characteristics, resection margins, and subsequent surgical decision-making. The timeliness and accuracy of frozen-section results directly influence the quality and safety of intraoperative clinical decisions (1). However, the frozen-section workflow involves multiple disciplines and sequential processes, including specimen acquisition, identification, documentation, transport, and pathological evaluation. Because of its time-sensitive nature and high degree of interdependence among different personnel and departments, any failure at a single step may result in diagnostic delays, specimen misidentification, inaccurate pathological interpretation, and inappropriate surgical management (2). Before implementation, frozen-section and routine pathology specimens were processed through largely similar manual workflows. Unlike routine specimens, frozen-section specimens require immediate identification, rapid transport, and timely pathological feedback during surgery. However, insufficient differentiation between these pathways could result in delayed transport, incomplete documentation, or incorrect prioritization, thereby affecting intraoperative decision-making. Previous studies have demonstrated that surgical specimen management errors predominantly occur during the pre-analytical phase, particularly during specimen submission and transfer, including incomplete requisition information, labeling discrepancies, patient identification errors, and transport delays. These failures may compromise the timeliness and reliability of pathological diagnosis, interfere with intraoperative decision-making, and ultimately threaten patient safety (3, 4, 8, 9).

Therefore, establishing a closed-loop specimen workflow that enables standardized identification, traceable handover, and real-time monitoring across the operating room and pathology department is essential for reducing preventable failures. Currently, rapid specimen management in many domestic hospital operating rooms remains largely dependent on manual processes, which are associated with several challenges. First, information transmission is vulnerable to delays and inaccuracies. Specimen-related information is frequently recorded manually, increasing the risk of errors in specimen identification, anatomical site, and labeling. In addition, incomplete documentation during critical steps, including specimen collection, transportation, and handover, limits end-to-end traceability throughout the workflow (5). Second, interdepartmental coordination remains insufficient because of the lack of effective information-sharing mechanisms among operating rooms, pathology departments, and clinical units. Without real-time monitoring during specimen transportation, delays, misdeliveries, and missed transfers may occur, potentially affecting the efficiency and reliability of pathological diagnosis (6). Third, risk management within specimen workflows remains inadequate. Potential failure modes are not systematically identified and prioritized, and targeted preventive strategies for high-risk steps are often lacking, which may contribute to specimen-related adverse events (6, 7).

Failure mode and effects analysis (FMEA) is a proactive quality improvement and risk management approach that systematically identifies potential failure modes, evaluates their possible effects, and prioritizes risks according to severity, occurrence, and detectability (10–13). By quantifying risk priority and implementing targeted corrective measures, FMEA facilitates a transition from reactive problem-solving to proactive prevention, thereby improving process reliability and safety. In this study, a multidisciplinary team applied FMEA, combined with an information-based closed-loop tracking system, to analyze the intraoperative rapid pathology specimen management workflow. High-risk failure modes were identified and prioritized, corresponding improvement interventions were developed, and continuous monitoring mechanisms were established to enhance specimen traceability, workflow efficiency, and management reliability (11).

## Aim

The aim of this study was to evaluate the effectiveness of an FMEA-guided digital redesign program for intraoperative rapid pathology specimen management. The program integrated a dedicated frozen-section pathway, standardized verification procedures, barcode-based handover, repeat-submission registration, role-specific staff training, and real-time monitoring of workflow deviations. The association between program implementation and improvements in transport timeliness, handover-record accuracy, and staff-reported process satisfaction was subsequently assessed.

## Methods

This single-center, non-randomized before-and-after service improvement study was conducted in the operating theaters and pathology department of an anonymized tertiary teaching hospital in eastern China. The study aimed to evaluate an FMEA-guided digital redesign program for intraoperative rapid pathology specimen management. The intervention was implemented as a workflow improvement initiative involving the establishment of a dedicated frozen-section pathway, standardized specimen verification procedures, barcode-based handover, repeat-submission registration, role-specific staff training, and real-time monitoring of workflow deviations.

The study consisted of two periods: a pre-implementation period from October to December 2025 and a post-implementation period from January to March 2026. Frozen-section specimens transferred during these periods were compared to assess changes in process-related outcomes after implementation. Reporting followed the Standards for QUality Improvement Reporting Excellence (SQUIRE) 2.0 guidelines (14).

### Service context

During the study periods, the service performed approximately 20,000 operations annually and processed approximately 80 frozen-section submissions per month. Specimens were transported by dedicated personnel from the operating theaters to the pathology department located on the same floor. Before implementation, specimen management relied on a combination of information systems, barcode processes, and paper-based records. However, staff identification, handover information, and specimen receipt timestamps were not integrated into a unified digital workflow, resulting in limited traceability across the specimen transfer process. Institutional policy required frozen-section specimens to be delivered to the pathology department within 5 min after handover. No other major workflow-wide changes were introduced during the study period.

A multidisciplinary team consisting of perioperative nursing leaders and staff, a surgeon, a pathology technologist, and an information technology engineer was established to redesign the workflow. The team mapped the entire specimen management pathway from request initiation to communication of pathology results and systematically reviewed potential risks related to clinical procedures, documentation, specimen identification, handover, and transportation. The identified risks were subsequently analyzed using FMEA to prioritize high-risk failure modes and guide targeted improvement strategies.

### Risk identification and prioritization

Potential failure modes and contributory factors were identified through systematic pathway review and structured multidisciplinary brainstorming. The analysis covered the entire frozen-section specimen workflow, including specimen request, identification, documentation, handover, transportation, and pathology receipt. For each failure mode, potential effects, contributing factors, and existing detection approaches were evaluated.

Severity (S), occurrence (O), and detectability (D) were independently scored from 1 to 10 according to locally agreed criteria. Severity reflected the potential impact of a failure on specimen safety, diagnostic accuracy, and intraoperative decision-making. Occurrence represented the frequency of the failure under routine practice conditions, whereas detectability indicated the likelihood that the failure could be identified before reaching the next process step or causing harm. Higher scores indicated greater severity, higher occurrence, or poorer detectability.

RPN is the product of the frequency of occurrence (O), detection rate (D), and severity (S) of the event. O, D, and S are each assigned values ranging from 1 to 10. O represents the likelihood of the failure mode occurring, with 1 indicating an extremely unlikely occurrence and 10 indicating a very likely occurrence; D represents the likelihood of the failure mode being detected, with 1 indicating a very likely detection and 10 indicating a less likely detection. Faults that are less likely to be detected require a higher priority because they are easily overlooked; S represents the severity of the consequences of the event failure mode, with 1 indicating a very small consequence and 10 indicating a very large consequence. The group members assign values to each failure mode based on their experience and calculate the RPN value. A higher RPN value indicates a higher risk level in the process, and it is more worthy of attention and should be given priority for control measures to be implemented for intervention. For the failure modes that occur in the process, the group members identify the top 4 failure modes with the highest RPN values (Table 1).

**Table 1:** Priority failure modes, risk assessment, and linked interventions identified by FMEA.

Failure mode	Potential effect	Contributing factors	Existing detection method	S	O	D	RPN	Linked intervention
Incomplete handover record or mismatch between requisition and specimen label	Delayed specimen identification, incorrect pathological processing, and impaired traceability	Manual documentation, inconsistent verification procedures, and incomplete handover information	Manual review of paper records and requisition-label comparison	9	5	7	315	Dedicated pathway; single-record repeat-submission register; barcode- and QR code-based handover
Delayed transport	Delayed frozen-section diagnosis and interruption of intraoperative decision-making	Lack of real-time transport monitoring; unclear responsibility for transfer timing	Retrospective review of delivery records	8	6	6	288	Dedicated scanner and handover point; automatic timestamps; live monitoring; escalation and retrospective review
Incorrect specimen name, collection site, or laterality	Misinterpretation of pathological information and inappropriate surgical decisions	Inadequate verification before submission; reliance on manual identification	Verbal confirmation and manual label checking	10	4	7	280	Standardized verbal verification; role-specific training; requisition-label concordance checks
Omitted, incorrect, or duplicate label	Specimen misidentification, delayed processing, or inability to match the specimen with patient information	Multiple specimens handled simultaneously; insufficient labeling control	Manual label inspection before transport	10	4	7	280	One-specimen-at-a-time verification and scanning; barcode controls; repeat-submission subcodes

### Intervention

Five interconnected changes were implemented as part of the FMEA-guided digital redesign program. First, a dedicated frozen-section pathway was established to distinguish time-critical frozen-section specimens from routine pathology specimens, with clearly defined responsibilities, verification procedures, and transport time requirements. Second, a redesigned registration process was introduced for repeat submissions from the same patient, in which all related specimens were recorded within a single record using sequential subcodes and specimen counts to improve traceability.

The intervention was structured around five critical points along the specimen pathway: (1) specimen identification and verification, (2) repeat-submission registration, (3) handover confirmation, (4) transport monitoring, and (5) continuous quality feedback. A workflow diagram was developed to illustrate the differences between the pre-implementation and post-implementation processes (Figures 1A,B).

**Figure 1:**
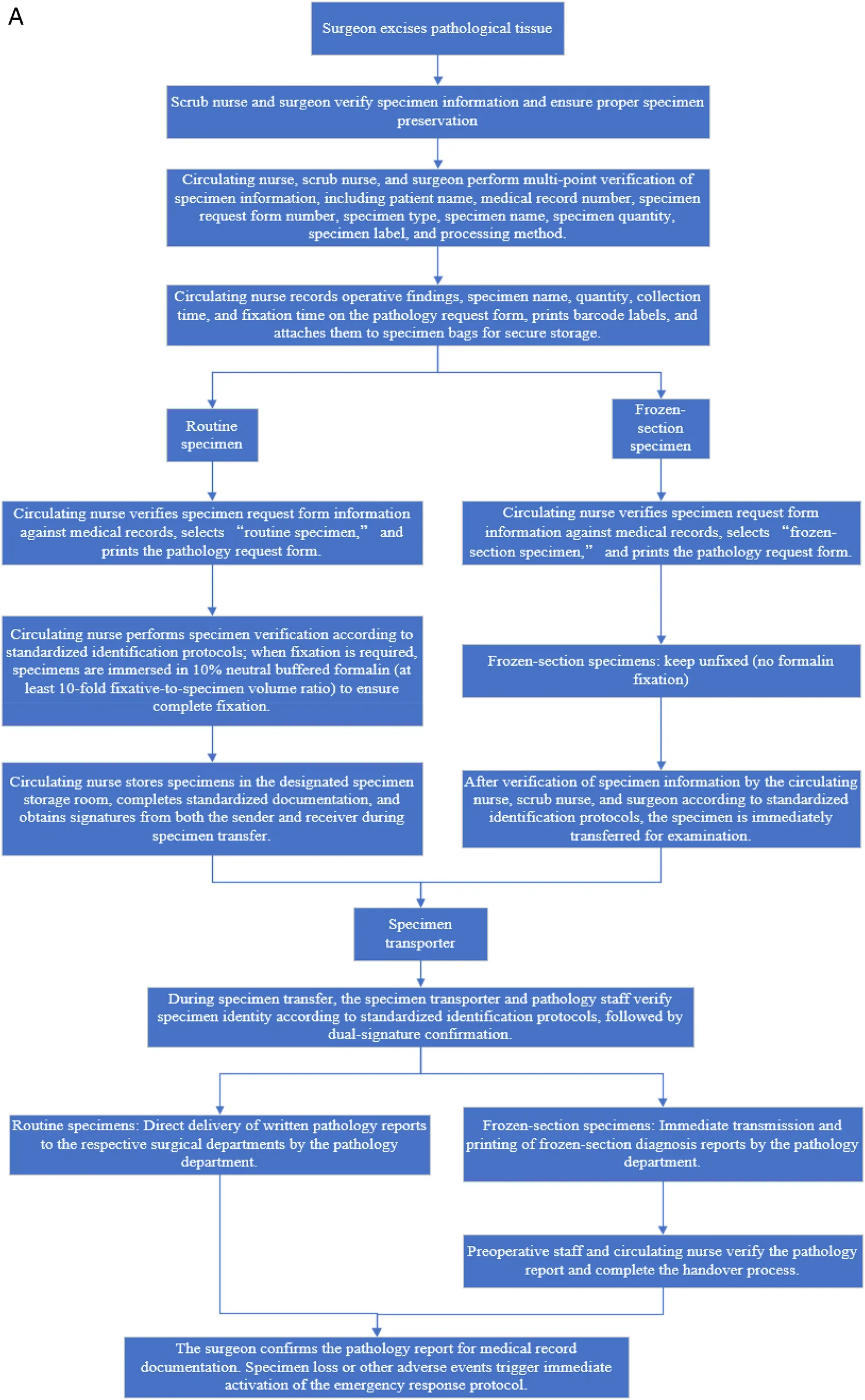
**(A)** A pre-implementation workflow. Created by the Department of Anesthesiology and Perioperative Medicine, Shandong First Medical University Affiliated Provincial Hospital, and is used with permission. **(B)** A post-implementation workflow.

**Figure:**
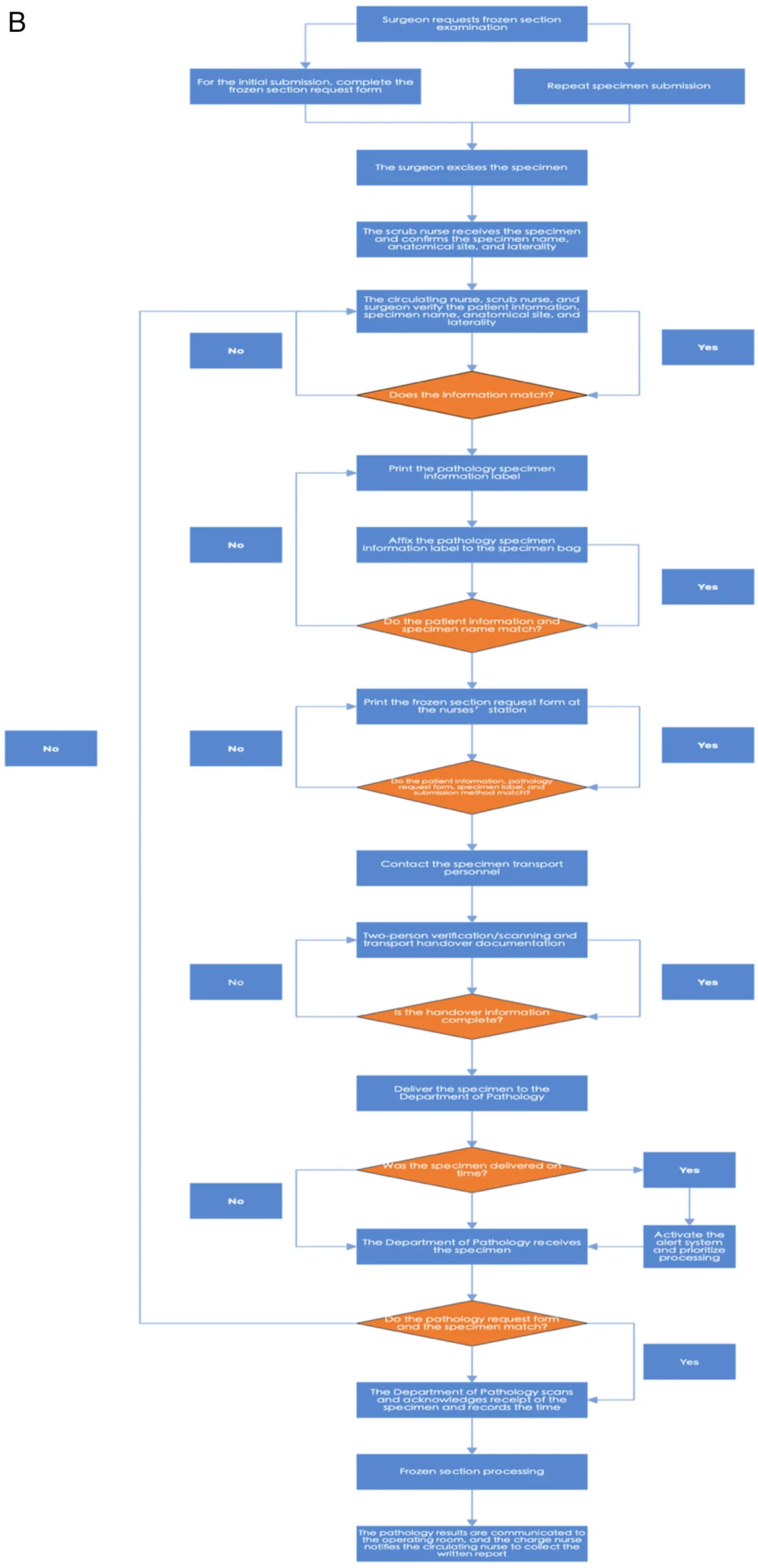


At the designated handover point, a computer and barcode scanner were used to capture specimen barcodes, transporter QR codes, and handover timestamps. Pathology receipt was confirmed through barcode scanning at the receiving point. The combination of manual verification, electronic timestamps, and digital signatures established a three-party traceable handover process involving operating-room staff, transport personnel, and pathology staff (Figures 2A–C).

**Figure 2:**
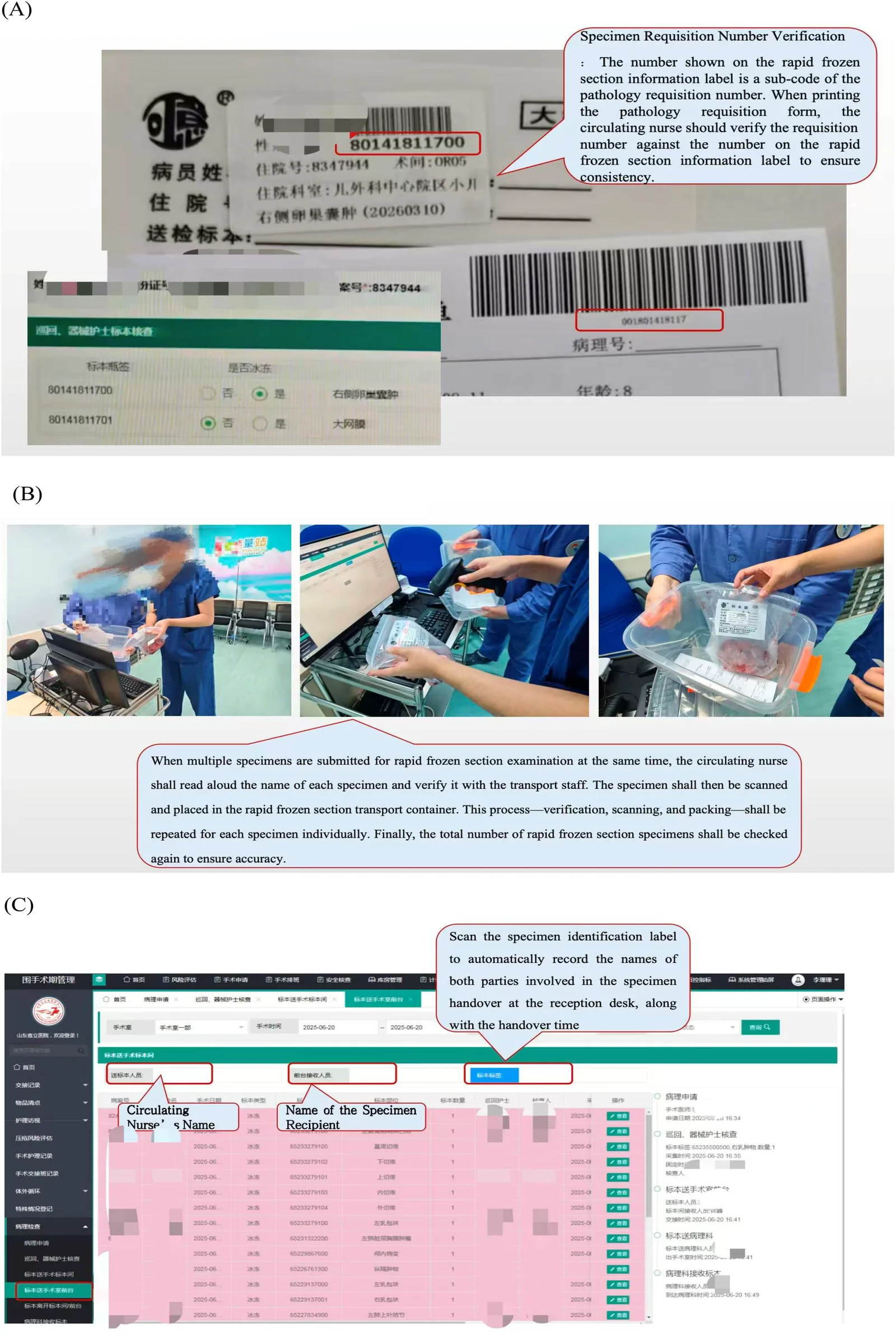
**(A)** Standardized specimen labeling. **(B)** Barcode-based handover confirmation. **(C)** Transport tracking and timestamp recording.

Role-specific training was provided before implementation and included workflow principles, specimen identification and verification procedures, barcode scanning operation, repeat-submission registration, and management of delayed or discrepant handovers. Training methods included brief lectures, demonstrations, simulation exercises, case-based discussions, and supervised system practice. Staff competency was assessed through simulated specimen-transfer scenarios before routine application. After implementation, circulating nurses monitored real-time workflow status and intervened when delays or discrepancies were identified, while a quality-control nurse performed weekly audits and provided feedback. The program was launched on 1 January 2026 following completion of training and supervised testing.

### Outcome measures and data collection

All registered frozen-section specimens during the study periods were included, with no additional exclusion criteria. The analyzed observations were individual frozen-section specimen transfers to the pathology department, including 238 specimens before implementation and 247 specimens after implementation. Multiple specimens from the same patient were retained and analyzed as separate specimen-transfer events.

The primary process outcomes were transport timeliness and handover-record accuracy. Transport timeliness was defined as the proportion of frozen-section specimens that reached pathology scanning or registration within 5 min after handover from the operating theater. The 5-min threshold was based on the institutional requirement for frozen-section specimen delivery and reflected the time-sensitive nature of intraoperative pathological decision-making.

Handover-record accuracy was defined as the proportion of specimen transfers with complete and consistent documentation, including patient identifiers, specimen identifiers, agreement between requisition information and specimen labels, specimen sequence number, responsible personnel, and transfer timestamps. This indicator was designed to assess the completeness and reliability of information capture required for end-to-end specimen traceability.

Staff-reported process satisfaction was assessed using a binary satisfied/not satisfied item completed by the same 62 staff members before and after implementation.

Pre-implementation data were obtained from paper registers and audit checklists, whereas post-implementation data were extracted from the barcode system, paper registers, and audit checklists. The same outcome definitions and assessment criteria were applied during both periods. A trained quality-control nurse reviewed all records. Data completeness was confirmed, and no specimens were excluded because of missing outcome data.

### Statistical analysis

Statistical analyses were performed using SPSS software (version 21). Continuous variables were expressed as mean ± standard deviation, and categorical variables were presented as frequencies and percentages. Because the primary outcomes were categorical process indicators, proportions were compared using chi-square tests or Fisher's exact tests, with risk ratios and 95% confidence intervals (CI) calculated to estimate effect size. A two-sided *p*-value <0.05 was considered statistically significant. Risk ratios and risk differences with 95% confidence intervals were calculated to describe the magnitude of change between the two periods.

## Results

### Process outcomes

During the pre-implementation period, 238 frozen-section specimens were included, compared with 247 specimens during the post-implementation period. According to the predefined outcome criteria, transport timeliness improved from 80.7% (192/238) before implementation to 98.4% (243/247) after implementation, representing an absolute increase of 17.7 percentage points (95% CI, 12.5–23.0) and a risk ratio of 1.22 (95% CI, 1.14–1.30; *p* < 0.001).

Handover-record accuracy increased from 75.2% (179/238) before implementation to 99.2% (245/247) after implementation, corresponding to an absolute increase of 24.0 percentage points (95% CI, 18.4–29.6) and a risk ratio of 1.32 (95% CI, 1.23–1.42; *p* < 0.001).

Staff-reported process satisfaction increased descriptively from 71.0% (44/62) before implementation to 95.2% (59/62) after implementation. Because individual paired responses were unavailable, this outcome was reported descriptively without inferential testing. (Table 2, Figure 3).

**Table 2:** Process outcomes before and after implementation of the FMEA-guided digital redesign program.

Outcome	Before implementation	After implementation	Absolute difference, after minus before (95% CI)	Risk ratio, after versus before (95% CI)	*p*-value
Transport timeliness	192/238 (80.7%)	243/247 (98.4%)	+17.7 percentage points (12.5–23.0)	1.22 (1.14–1.30)	<0.001
Handover-record accuracy	179/238 (75.2%)	245/247 (99.2%)	+24.0 percentage points (18.4–29.6)	1.32 (1.23–1.42)	<0.001
Staff-reported process satisfaction	44/62 (71.0%)	59/62 (95.2%)	+24.2 percentage points	Not calculated	—

**Figure 3:**
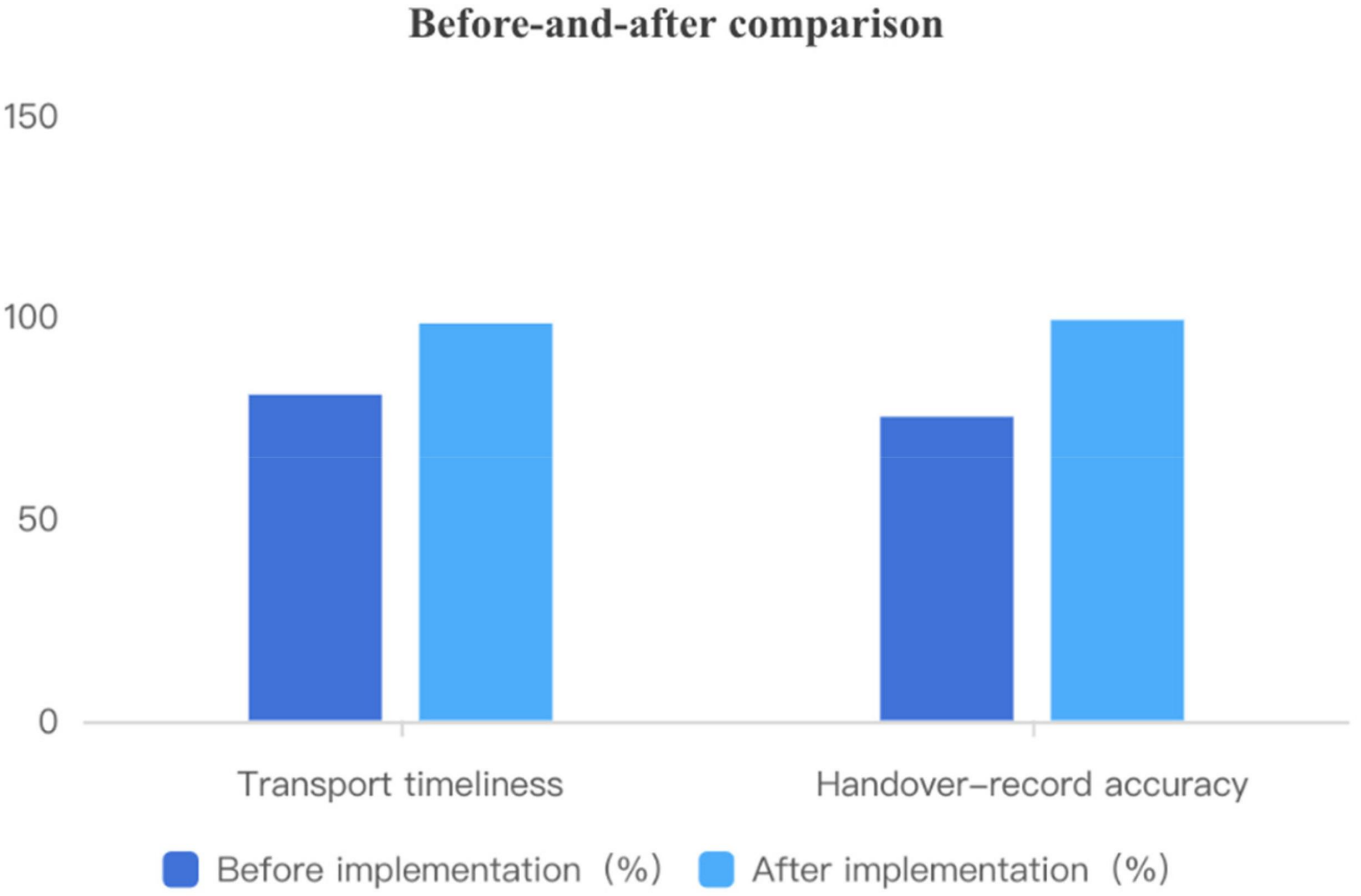
A comparison of process outcomes before and after implementation of the FMEA-guided digital redesign program.

## Discussion

This study integrated FMEA with an information-based closed-loop tracking system to redesign the intraoperative frozen-section specimen pathway. By identifying and prioritizing high-risk failure modes, targeted interventions were developed for critical workflow steps, including specimen identification, repeat-submission registration, handover confirmation, transport monitoring, and continuous quality feedback. Following implementation of the redesigned pathway, improvements were observed in transport timeliness, handover-record accuracy, and staff-reported process satisfaction, suggesting that an FMEA-guided digital redesign approach may enhance specimen traceability and workflow reliability.

The combination of FMEA and digital closed-loop management created a risk-oriented improvement model. FMEA guided the selection of intervention priorities by identifying the most critical failure modes, whereas the digital system supported standardized implementation through automated data capture, traceable handover, and continuous monitoring. In this study, workflow monitoring and regular quality review provided ongoing feedback for process optimization, transforming specimen management from reactive correction toward proactive prevention and continuous improvement (15).

The observed improvement in transport timeliness may be related to several coordinated changes, including the establishment of a dedicated frozen-section pathway, clearly defined handover responsibilities, automatic timestamp recording, and real-time workflow monitoring. Similarly, the increase in handover-record accuracy may be associated with barcode verification, standardized documentation requirements, and three-party confirmation involving operating-room staff, transport personnel, and pathology staff. These findings highlight that improving specimen safety requires not only technological support but also standardized procedures, clear accountability, and multidisciplinary collaboration.

### Limitations

This study has several limitations. First, the single-center before-and-after design limits the ability to establish causal relationships, and the observed improvements may have been influenced by temporal trends, increased staff awareness, or other unmeasured factors. Second, because multiple intervention components were implemented simultaneously, the independent contribution of each component could not be determined. Third, this study primarily evaluated process outcomes, including transport timeliness and handover-record accuracy, and did not assess longer-term clinical outcomes such as diagnostic turnaround time, specimen-related errors, frozen-section diagnostic concordance, or patient outcomes. Finally, the sustainability and generalizability of this redesigned workflow require further validation through multicenter studies and longer follow-up periods.

## Conclusion

An FMEA-guided digital closed-loop redesign program was associated with improvements in transport timeliness, handover-record accuracy, and traceability within the intraoperative frozen-section specimen pathway. The approach of integrating proactive risk identification with standardized digital process control provides a feasible strategy for optimizing perioperative specimen management, strengthening multidisciplinary coordination, and supporting continuous quality improvement.

## Data Availability

The raw data supporting the conclusions of this article will be made available by the authors, without undue reservation.
